# Isolation and Molecular Identification of Potentially Pathogenic Free‐Living Amoeba in Tap Water Faucets in Quezon City, Philippines

**DOI:** 10.1155/japr/8388296

**Published:** 2026-06-09

**Authors:** Jehudiel Eugenio R. Tiglao, Davin Edric V. Adao

**Affiliations:** ^1^ Pathogen–Host–Environment Interactions Research Laboratory, Institute of Biology, College of Science, University of the Philippines Diliman, Quezon City, Metro Manila, Philippines, upd.edu.ph; ^2^ Natural Sciences Research Institute, University of the Philippines Diliman, Quezon City, Metro Manila, Philippines, upd.edu.ph

**Keywords:** free-living amoebae, isolation, molecular identification, Philippines, tap water

## Abstract

Free‐living amoebae (FLA) are ubiquitous in environmental water and soil, and their presence in water systems for human consumption is a public health concern due to their potential pathogenicity and ability to act as a reservoir for pathogenic bacteria. This study identified potentially pathogenic FLAs in urban water systems, specifically from tap water faucets. A total of 26 faucets were sampled, with 16 (62%) testing positive for FLAs. These included faucets from comfort rooms (*n* = 6), laboratories (*n* = 4), households (*n* = 14), and public parks (*n* = 2). Initial identification was based on the morphology of trophozoites and cysts, followed by molecular identification using universal 18S rRNA primers and sequencing. A total of 21 FLAs were isolated, including 11 *Vermamoeba vermiformis*, three *Vannella* spp., and one each of *Ptolemeba bulliensis*, *Protacanthamoeba bohemica*, *Stenamoeba polymorpha*, *Stenamoeba amazonica*, and *Micriamoeba tesseris*, along with two unidentified species. One of the unidentified isolates exhibited branching trophozoites and distinct double‐walled cysts. The high detection rate of FLAs in tap water suggests a potential health risk, either through direct infection or by serving as vectors for pathogenic bacteria. These findings highlight the importance of routinely monitoring urban water systems for FLA contamination to safeguard public health.

## 1. Introduction

Free‐living amoebae (FLA) are a polyphyletic group of amoeboid protozoa that are ubiquitously found in environmental soil and water. However, these organisms can contaminate water systems and have been isolated in various sources, such as tap water [1], dental units [2, 3], swimming pools [4], thermal springs [5], and shower units [6].

Some species of FLAs are known to cause disease in humans, with clinical manifestations ranging from skin lesions and severe ocular infections to fatal infections of the central nervous system (CNS). Traditionally, cases of FLA infections have been documented in four primary genera. Species within the genus *Acanthamoeba*, for example, are the causative agents of *Acanthamoeba* keratitis (AK), a severe eye infection often associated with contact lens use, as well as granulomatous amoebic encephalitis (GAE), a rare but typically fatal CNS disease. *Acanthamoeba* is also known to cause severe cutaneous lesions (reviewed in [7]). Other genera, including *Naegleria* [8], *Balamuthia* (reviewed in [9]), and *Sappinia* [10], are primarily associated with high‐mortality brain infections, though *Balamuthia* may also present with skin lesions. However, recent studies have discovered that other FLA species, specifically *Vermamoeba vermiformis*, can also cause infections in humans or contribute to the progression of disease ([11]; [12–13]). This suggests that FLA infections are not limited to the four studied genera, and other FLA species can be potentially pathogenic.

Additionally, FLAs have been shown to harbor pathogenic microorganisms, serving as protective barriers or “Trojan horses” that could facilitate the invasion and spread of these pathogens [14–15]. Studies have also explored the role of FLAs as a “training ground” for microorganisms, increasing their virulence or causing the emergence of pathogenicity in bacteria [16–17] and fungi [18, 19].

Despite the public health risks, either through causing diseases or by acting as reservoirs for pathogenic microbes, studies on FLAs remain limited. To our knowledge, only three genera of FLA have been isolated and studied in the Philippines. These include *Acanthamoeba*, isolated from soil, water, and contact lens cases [20, 21], nasal swabs [22], and water reservoirs [23]; *V. vermiformis* isolated from a freshwater fish [24]; and *Naegleria australiensis* isolated from a lake [25].

This study is aimed at identifying FLA species present in tap water faucets, which serve as a primary source of household water and may act as vehicles for these organisms. Recognizing potentially pathogenic FLAs is vital for public health. Currently, there are no national protocols for detecting protozoan parasites in water in the Philippines, with monitoring limited only to fecal coliform [26]. Therefore, this study contributes to understanding FLA distribution in urban water systems and highlights the associated health risks.

## 2. Materials and Methods

### 2.1. Sampling and Sample Processing

A total of 26 water faucets from households (*n* = 14), laboratories (*n* = 4), comfort rooms (*n* = 6), and a public park (*n* = 2) were sampled using sterile cotton swabs (Table 1). The swabs were placed in a sterile 15‐mL conical tube and transported to the laboratory immediately for processing. Swab samples were washed thoroughly by adding 4 mL of sterile Page′s *Acanthamoeba* saline (PAS), and the swabs were rubbed on a 1.5% Page′s *Acanthamoeba* saline‐nonnutrient agar (PAS‐NNA) seeded with *Escherichia coli* (ATCC 25922). The solution was centrifuged at 1500 rpm for 10 min to collect the debris. The pellet (200 *μ*L) was inoculated on a separate PAS‐NNA–*E. coli* plate. Plates were incubated at 30°C and observed daily for up to 14 days for FLA growth before being considered negative. Initial identification of FLAs was based on trophozoite and cyst morphology [27]. Morphological examination was performed using a light compound microscope (Optika, Italy). Distinct morphologies were isolated by cutting a piece of agar containing isolated cysts or trophozoites and transferring it face down onto a fresh PAS‐NNA–*E. coli* plate. This process was repeated until an isolate was obtained and grown as a homogeneous culture.

**Table 1:** Number of processed samples, culture‐positive samples, and distinct FLA isolates from water faucets in various selected sources in Metro Manila, Philippines.

Sampling source	Number of samples	Culture‐positive samples number (%)	Number of distinct FLA isolates
Household (HH)	14	8 (57.1%)	12
Public comfort room (CR)	6	4 (66.6%)	4
Public parks (PP)	2	1 (50.0%)	1
Laboratory (LR)	4	3 (75.0%)	4
Total	26	16 (61.5%)	21

### 2.2. DNA Extraction and Polymerase Chain Reaction (PCR)

After obtaining a homogeneous culture, DNA extraction was performed using Chelex resin (Chelex‐100, Sigma‐Aldrich, Saint Louis, Missouri, United States) following the protocol established by Iovieno et al. [28]. Briefly, cells from the plates were collected by flooding the plates with phosphate‐buffered saline (PBS, pH 7.4) and scraping the cells using a sterile cell scraper. Cells were collected using a pipettor, transferred into 1.5‐mL microcentrifuge tubes, and centrifuged at 8,500 X g for 6 min. The centrifugation steps were conducted two more times for washing. Two hundred (200) *μ*L of Chelex solution (10% w/v Chelex‐100, 0.1% Triton X‐100, 10 mM Tris pH 8.0) were added to the cells, vortexed for 10 s, centrifuged at 10,000 × g for 10 s, heated at 95°C for 20 min, and centrifuged again at 10,000 × g for 20 s. One hundred fifty (150) *μ*L of the supernatant was collected and subjected to PCR or stored at −20°C.

PCR was performed using the T100 Thermal Cycler (Bio‐Rad Laboratories, Hercules, California, United States). The reaction mixture consisted of GoTaq G2 Master Mixes (Promega, Madison, Wisconsin, United States), with universal eukaryotic 18S rRNA primers Euk1A (5 ^′^‐CTGGTTGATCCTGCCAG‐3 ^′^) and Euk516r‐GCb (5 ^′^‐ACCAGACTTGCCCTCC‐3 ^′^) [29] under the following conditions: initial denaturation at 94°C for 5 min, followed by 30 cycles of denaturation at 94°C for 45 s, annealing at 52°C for 1 min, and extension at 72°C for 2 min, with a final extension at 72°C for 7 min. PCR amplicons were visualized using a 1.5% agarose gel, and the band size was determined using a DNA ladder (HyperLadder 100 bp: Meridian Bioscience, United States). In the case of sample HHFS‐4c, the primers Ami6F2/Ami9R [30] were used because primers Euk1A/Euk516r‐GCb failed to amplify the desired 18S rRNA segment. The following conditions were used: initial denaturation at 94°C for 5 min, followed by 30 cycles of denaturation at 94°C for 1 min, annealing at 55°C for 30 s, and extension at 72°C for 2 min. The final extension was at 72°C for 10 min. Amplicons yielded ~650 base pairs for both primer sets. Positive amplicons were sent to Macrogen (Seoul, South Korea) for purification and sequencing. A total of 21 amplicons were submitted, of which 19 were derived from individual isolates and were successfully identified. The remaining two amplicons originated from isolate CRFS‐338 using the primer sets Euk1A/Euk516r‐GCb and Ami6F2/Ami9R; however, these yielded poor‐quality, noninterpretable reads. Obtained sequences were processed using MEGA v. 11 [31] and subjected to a nucleotide similarity search using the National Institutes of Health′s Basic Local Alignment Search Tool (NCBI BLAST). Molecular identification of isolates, together with their reference sequences in the NCBI Core Nucleotide Database, is provided in the Supporting Information (Table S1).

### 2.3. Phylogenetic Analysis

Phylogenetic analysis of partial 18S rDNA sequences from both the isolates and reference FLAs was performed using MEGA v.12 [32] and MrBayes v.3.2.7a [33]. A complete list of reference sequences included in the analysis is provided in the Supporting Information (Table S2). Among the molecularly identified isolates, *M. tesseris* was excluded from the phylogenetic reconstruction due to the use of a different primer set for its identification, as it showed negative amplification with the universal Euk1A‐Euk516r‐GCb primers. All sequences were aligned using ClustalW within MEGA v.12.

The optimal model of DNA substitution was first determined using jModelTest v.2.1.10 [34], with model selection based on the Bayesian information criterion (BIC). Neighbor‐joining (NJ) and maximum likelihood (ML) phylogenetic trees were constructed using MEGA v.12, based on the Tamura 3‐parameter model with gamma distribution (T92 + G), which was selected as the best‐fit model by MEGA v.12. ML bootstrap support values were estimated using MEGA′s Adaptive Bootstrap feature to improve computational efficiency, whereas NJ trees were assessed with 1,000 bootstrap replicates. Bayesian inference (BI) was performed using MrBayes v.3.2.7a, under the Kimura 2‐parameter model with gamma distribution (K80 + G) as identified by jModelTest v. 2.1.10. Two independent runs of four Markov chains were conducted for 1 million generations, sampling every 1,000 generations, and convergence was assessed by monitoring the average standard deviation of split frequencies (< 0.01). The resulting BI tree was rooted to the Tubulinea group to separate it from the Discosea group, and only node support values ≥ 0.70 (BI posterior probability) and ≥ 50% (ML and NJ bootstrap) were considered significant. Final visualization and annotation of the consensus tree were performed using iTOL v.7 [35]. The sequences of the isolates have been deposited in GenBank under Accession Numbers PX102193 and PQ345822–PQ345839.

## 3. Results

Sixteen (16) of the 26 water faucets sampled showed positive growth for FLAs. In some instances, a single faucet sample yielded multiple FLA species based on morphological examination, resulting in a total of 21 distinct isolates recovered from the 16 positive water faucet samples (Table 1). Nineteen (19) of these isolates were molecularly identified, which include *V. vermiformis* (11), *Vannella* spp. (3), *Ptolemeba bulliensis* (1), *Protacanthamoeba bohemica* (1), *Stenamoeba polymorpha* (1), *Stenamoeba amazonica* (1), and *Micriamoeba tesseris* (1). *M. tesseris* did not amplify with the universal Euk1A/Euk516r‐GCb primers but was successfully identified using the Ami6F2/Ami9R primers [30]. Two isolates, HHFS‐4B and CRFS‐338, could not be identified using molecular methods due to amplification failure despite multiple attempts. The molecular identification of the isolates, along with their corresponding reference sequences from the NCBI Core Nucleotide Database, is provided in the Supporting Information (Table S1).

**Figure 1:**
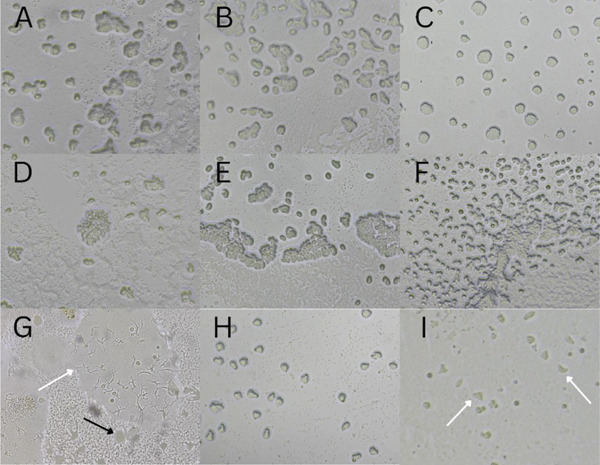
Morphological examination of FLAs on the surface of agar plates under 100× magnification: (A) *P. bulliensis* (HHFS‐2a), (B) unknown (HHFS‐4B), (C) *M. tesseris* (HHFS‐4c), (D) *S. polymorpha* (HHFS‐7a2), (E) *S. amazonica* (HHFS‐7b), (F) *V. vermiformis* (CRFS‐318), (G) unknown (CRFS‐338), (H) *Vannella* sp. (LFRS‐346b), and (I) *P. bohemica* (HHFS‐6b1). In Panel (G), the white arrow indicates the branching trophozoite of isolate CRFS‐338 within a gel‐like substance, whereas the black arrow denotes its bulbous form outside of it.

**Figure 2:**
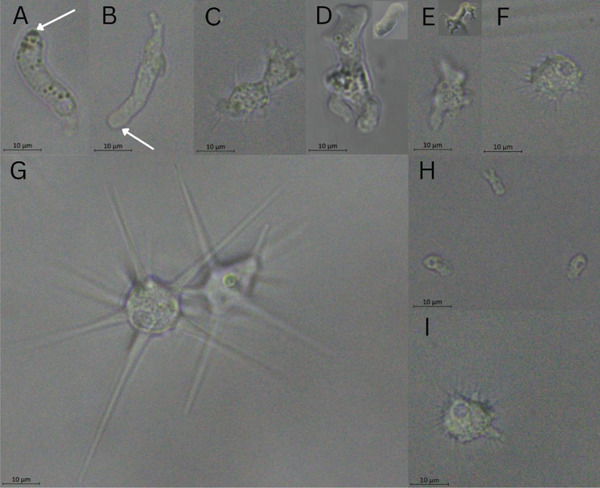
Floating form of isolates in PAS solution: (A) *P. bulliensis*, (B) *V. vermiformis*, (C) unknown (HHFS‐4B), (D) *S. polymorpha*, (E) *S. amazonica*, (F) *P. bohemica*, (G) *Vannella* sp., (H) *M. tesseris*, and (I) unknown (CRFS‐338). In Panel (B), the white arrow indicates the presence of a hyaline cap in *V. vermiformis*; this feature is absent in *P. bulliensis* (Panel A). Insets in the upper right of Panels (D) and (E) show the monotactic form of *S. polymorpha* and the distinct slender pseudopodia in the polytactic form of *S. amazonica*, respectively.

Figure 1 presents the morphological characteristics of the isolated FLAs, most of which appear in trophozoite form as they feed on *E. coli* atop nonnutrient agar plates. Distinct morphologies were observed, including small amoebae predominantly in cyst form (Figure 1C), branching trophozoites (Figure 1G), and a noncyst‐forming, fan‐shaped trophozoite (Figure 1H). Figure 2 shows the floating forms of these isolates, highlighting morphotypes specific to different genera. Initial descriptions were based on the criteria established by Smirnov and Brown [27], whereas further detailed characterization was conducted following species determination via 18S rRNA gene sequencing. *P. bulliensis* and *V. vermiformis* exhibit a monotactic morphotype, distinguished by the presence of a hyaline cap in *V. vermiformis* and its absence in *P. bulliensis* (Figure 2A,B, white arrow). Both *Stenamoeba* isolates display primarily a polytactic form, with long, slender pseudopodia, though they are also capable of assuming a monotactic shape. A polytactic form of *S. polymorpha* is shown in Figure 2D, whereas a monotactic form of *S. polymorpha* is shown in Figure 2D top‐right inset. In addition, a monotactic form of *S. amazonica* is shown in Figure 2E and its polytactic form in Figure 2E top‐right inset. *M. tesseris* is notably smaller than the other isolates and forms a lingulate morphotype (Figure 2H). *Vannella* spp. display a fan‐shaped morphotype on agar (Figure 1H); however, their floating form is distinct, characterized by long, pointed pseudopodia extending from a central body (Figure 2G). Isolate HHFS‐4B, an unidentified FLA, shows finger‐like hyaline subpseudopodia, indicative of a dactylopodial morphotype (Figure 2C). *P. bohemica* displays an acanthopodial morphotype, forming numerous short, sharp subpseudopodia radiating from the central body (Figure 2F). A similar morphotype is observed in isolate CRFS‐338, another unidentified FLA. However, morphological differences are evident on agar: *P. bohemica* forms small, irregular trophozoites (Figure 1I, white arrow), whereas CRFS‐338 displays long, slender, branching trophozoites within a gel‐like substance (Figure 1G, white arrow) and bulbous trophozoites outside of it (Figure 1G, black arrow). Despite repeated DNA extraction attempts and the use of various universal eukaryotic 18S rRNA primer sets, isolates HHFS‐4B and CRFS‐338 could not be molecularly identified. HHFS‐4B produced no amplification across all primer sets. CRFS‐338 yielded a positive PCR result using the ERIB1‐ERIB10 [36] and Ami6F2‐Ami9R [30] primers, but sequencing was unsuccessful due to poor‐quality, noninterpretable reads.

**Figure 3:**
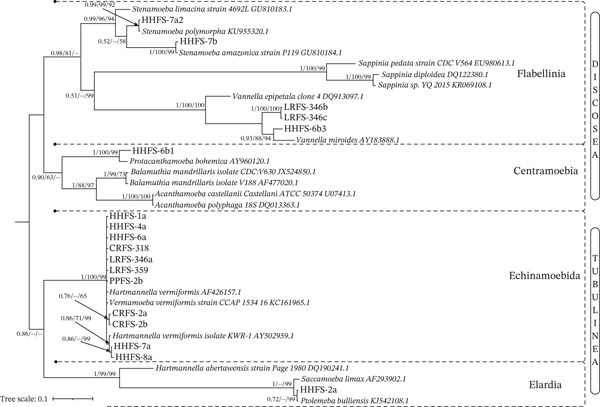
Consensus phylogenetic tree based on the Bayesian inference tree of FLAs isolated from tap water faucets, alongside reference sequences. Each node is annotated with three values: BI posterior probability and bootstrap support values from ML and NJ analyses, respectively. Support values above 0.70 (BI) and 50% (ML/NJ) were considered significant; however, values as low as 0.50 in BI are still shown. The scale bar indicates 0.1 substitutions per nucleotide position.

Phylogenetic analysis using BI, ML, and NJ methods consistently resolved the clustering of FLA isolates with reference sequences into well‐supported taxonomic groups (Figure 3). *V. vermiformis* isolates (CRFS‐318, CRFS‐2a, CRFS‐2b, PPFS‐2b, HHFS‐1a, HHFS‐4a, HHFS‐6a, HHFS‐7a, HHFS‐8a, LRFS‐346a, and LRFS‐359) grouped tightly with *V. vermiformis* reference sequences, forming a clade within the class Echinamoebida. *P. bulliensis* isolate (HHFS‐2a) clustered with *Saccamoeba* and *Hartmannella*, all members of the class Elardia. Both classes belong to the phylum Tubulinea. *P. bohemica* isolate (HHFS‐6b1) formed a cluster with *P. bohemica* reference sequence AY960120.1 and formed a group with the reference sequences of *Acanthamoeba* and *Balamuthia*, forming the class Centramoebia. In contrast, *Vannella* isolates (HHFS‐6b3, LRFS‐346b, and LRFS‐346c), *Stenamoeba* isolates (HHFS‐7a2 and HHFS‐7b), and *Sappinia* were placed within the class Flabellinia. The two classes, Centramoebia and Flabellinia, formed a monophyletic clade under the phylum Discosea. The overall tree topology, supported by high posterior probabilities and bootstrap values, confirms the molecular identities of the isolates and reflects the deep evolutionary divergence among major FLA lineages. These results are consistent with current taxonomic frameworks [37].

## 4. Discussion

Despite the known health risks associated with FLAs, studies on their distribution remain limited, particularly in the Philippines. Only a handful of investigations have examined their occurrence across various environments. Adao and Rivera [20] reported the presence and genotypes of *Acanthamoeba*, a medically significant FLA known to cause keratitis and GAE, in samples collected from contact lens cases, soil, and water. Genotyping revealed that most isolates belonged to the T4 and T5 genotypes, with both genotypes frequently recovered from contact lens cases [21]. Similarly, Cruz and Rivera [22] detected *Acanthamoeba* genotypes T4 and T5 in the human nasal cavity. Additional studies have also identified FLAs in man‐made aquatic environments. *Acanthamoeba* genotypes T3, T4, T5, and T11 were isolated from water reservoirs [23], and *N. australiensis* was detected in a major lake [25]. Moreover, FLAs have been identified in fish hosts, *Oreochromis niloticus*, with molecular identification confirming the presence of *V. vermiformis* [24].

Among the isolates identified in this study, *V. vermiformis* exhibited the highest prevalence, being detected in 11 out of 26 sampling sites (42%). This finding aligns with previous reports indicating that *V. vermiformis* is among the most frequently isolated FLA from various environments, including tap water [1], dental units [38], drinking water networks [39], and even in bottled mineral water [40]. The widespread presence of this species may be attributed to its ability to form highly resistant cysts and tolerance to high temperatures and chemical disinfectants [41, 42]. Although the pathogenicity of *V. vermiformis* remains uncertain, studies have explored its pathogenic potential. Kinnear [43] demonstrated its cytopathic effects on human corneal cells in vitro, producing similar destructive effects as *Acanthamoeba castellanii.* Furthermore, *V. vermiformis* was reported as the etiological agent of a painful ulcer near the eye after being isolated from a tissue infection in a female patient, suggesting potential pathogenicity [13]. This is further supported by a recent report confirming *V. vermiformis* as the etiological agent in a 48‐year‐old male with non‐acanthamoebic keratitis; the identification was confirmed by culturing the amoeba followed by DNA sequence analysis. Notably, no bacterial or fungal growth was detected, indicating that *V. vermiformis* was the sole etiological agent [44]. Additionally, recent animal studies have demonstrated that *V. vermiformis* can induce allergic airway inflammation in murine models [45]. Collectively, these findings highlight the need for further investigation into the pathogenic potential of this amoeba, as its high prevalence in various environments may pose significant public health risks.

Several other cyst‐forming species were also isolated in this study, including *S. polymorpha*, *S. amazonica*, *P. bohemica*, and *M. tesseris*, as well as one unidentified amoeba with an *Acanthamoeba*‐like floating form trophozoite. These genera remain relatively understudied compared with other FLAs. *S. amazonica* was first isolated from the gill tissue of a freshwater fish (*Psectogaster amazonica*) in the Amazon River, Peru [46] and has not been reported since; thus, this study represents the first report of this species since its initial isolation. On the other hand, *S. polymorpha* was first isolated from the diarrheic stool of a horse [47] and was also recently recovered from park water supplies in Arak, Iran [48]. To date, only seven species within the *Stenamoeba* genus have been formally described, with *Stenamoeba aeronauta* being the most recently identified [49]. This species was compared with other *Stenamoeba* species and delineated based on differences in their 18S rRNA gene sequences. Another species, *Stenamoeba dejonckheerei*, was isolated from a thermal hot spring with a temperature of 45.5°C, suggesting that members of this genus may exhibit thermotolerance. However, their potential pathogenicity and associations with bacterial endosymbionts remain poorly understood. Moreover, difficulties in achieving axenic cultivation, as observed with *S. dejonckheerei*, have hindered further experimental studies to assess their pathogenic potential. *P. bohemica* was first isolated from the liver of the fish *Tinca tinca* [50]. Since its initial discovery, it has also been recovered from swimming pools [51], various hospital environments [52], and drinking water distribution systems [39]. Although it shares some morphological characteristics with *Acanthamoeba*, the pathogenic potential of *P. bohemica* remains largely unexplored. Notably, *P. bohemica*, along with other FLA such as *Acanthamoeba*, *Vermamoeba*, and *Echinamoeba*, has been shown to harbor *Mycobacterium* species, particularly *Mycobacterium llatzerense* [39]. This association may offer a protective niche for these opportunistic pathogens and facilitate their replication. In contrast, *M. tesseris* was first described in 2012 and isolated from cooling towers and soil samples. Phylogenetic analyses place it closely related to *Echinamoeba* and *Vermamoeba*. Interestingly, unlike its close relatives, *M. tesseris* has been found not to internalize *Legionella pneumophila*, suggesting potential differences in host‐pathogen interactions within this group [53].

Noncyst‐forming FLAs, such as *Vannella* and *Ptolemeba*, were also isolated in this study. *Vannella* has been reported in a variety of environments, and numerous species have been described to date [54–55]. This genus is regarded as one of the most widespread amoebae across various habitats, yet it remains among the most difficult to differentiate at the species level [27]. In this study, all three *Vannella* isolates were observed to be noncyst‐forming; however, the genus includes species capable of forming cysts, such as *Vannella persistens* [56] and *Vannella pentlandii* [57]. *Vannella* species are frequently recovered from anthropogenic water systems. For instance, they have been isolated from toilet cistern storage tanks [58], and direct 18S rDNA sequencing has revealed their persistence in chlorinated finished drinking water from treatment plants [59]. Furthermore, *Vannella* spp. have been identified within hospital biofilms [60], highlighting their ability to colonize sensitive healthcare environments. In addition to their environmental prevalence, members of this genus may play a role in human health by harboring pathogenic parasites. Hoffmann et al. [61] first observed this “Trojan horse” phenomenon in *Vannella cirrifera* isolated from a potable warm‐water system, which supported the intracellular multiplication of microsporidia. This potential pathogenic role was further highlighted when *Vannella* was isolated from the eye and contact lens case of a female patient with keratitis; notably, these isolates also harbored microsporidia‐like microorganisms [62]. *Ptolemeba* is a relatively novel genus, previously classified under *Saccamoeba*. It was first isolated in 2014 from a soil sample [63] and has also been recovered from a rainbow trout affected by nodular gill disease, which demonstrated the ability to tolerate salinities of up to 25% [64]. Although the endosymbiotic associations and pathogenicity of *Ptolemeba* remain unstudied, its close relative *Saccamoeba* has been shown to exclusively harbor a chlamydia‐like parasitic endosymbiont [65], suggesting that *Ptolemeba* may exhibit similar biological interactions. As noncyst‐forming FLAs, the isolated *Vannella* spp. and *P. bulliensis* exist solely in the trophozoite stage. This suggests that trophozoites alone may be capable of withstanding basic water decontamination procedures, raising concerns about their potential to contaminate treated water supplies.

To our knowledge, this study represents the first report of the isolation of *M. tesseris* and *S. amazonica* since their initial discoveries in 2014 and 2010, respectively. Additionally, this research provides the first formal identification of *P. bulliensis*, *S. polymorpha*, and *Vannella* spp. within the Philippines. Despite the potential health risks posed by these organisms, there are currently no national guidelines in the Philippines for monitoring protozoan parasites in water systems, as existing standards focus only on fecal coliform. This gap highlights the need for more research on the presence and possible health impacts of FLAs. Moreover, many households in urban areas rely on untreated tap water for daily use, often without filtration, which may increase exposure. Given how little is known about these organisms, their role in waterborne diseases may be underestimated.

## Funding

This study was supported by Natural Sciences Research Institute, University of the Philippines (10.13039/501100011894; BIO‐24‐1‐04).

## Conflicts of Interest

The authors declare no conflicts of interest.

## Supporting Information

Additional supporting information can be found online in the Supporting Information section.

## Supporting information


**Supporting Information 1** Table S1: List of isolates and their corresponding reference sequence from the NCBI′s Core Nucleotide Database.

**Supporting Information 2** Table S2: Reference FLA sequences used in phylogenetic analyses, retrieved from the NCBI Core Nucleotide Database.

## Data Availability

The data that support the findings of this study are available from the corresponding author upon reasonable request.
