# Supplementary material for: Isolation and Molecular Identification of Potentially Pathogenic Free‐Living Amoeba in Tap Water Faucets in Quezon City, Philippines
Source: J Parasitol Res. 2026 Jun 9;2026:8388296. doi: 10.1155/japr/8388296 (PMC13248780; doi:10.1155/japr/8388296)
Supplement: Supplementary file 1 — Supporting Information 1 Table S1: List of isolates and their corresponding reference sequence from the NCBI′s Core Nucleotide Database. [file JAPR-2026-8388296-s002.pdf]

TABLE S1. List of isolates and their corresponding reference sequence from the NCBI's core nucleotide database

Isolate	Sampling source	Reference sequence in the NCBI's Core Nucleotide Database	GenBank Accession No.	Percent Identity	Coverage (%)
CRFS-318	Comfort room	*Vermamoeba vermiformis* strain CCAP 1534/17 18S ribosomal RNA gene, partial sequence (KC188996.1)	PQ345826	100.00	100
CRFS-2a	Comfort room	*Vermamoeba vermiformis* strain CCAP 1534/17 18S ribosomal RNA gene, partial sequence (KC188996.1)	PQ345825	99.63	100
CRFS-2b	Comfort room	*Vermamoeba vermiformis* strain CCAP 1534/17 18S ribosomal RNA gene, partial sequence (KC188996.1)	PQ345839	99.63	100
PPFS-2b	Public park	*Vermamoeba vermiformis* strain CCAP 1534/17 18S ribosomal RNA gene, partial sequence (KC188996.1)	PQ345822	99.63	100
HHFS-1a	Household	*Vermamoeba vermiformis* strain CCAP 1534/17 18S ribosomal RNA gene, partial sequence (KC188996.1)	PQ345833	100	100
HHFS-4a	Household	*Vermamoeba vermiformis* strain CCAP 1534/17 18S ribosomal RNA gene, partial sequence (KC188996.1)	PQ345832	100	100
HHFS-6a	Household	*Vermamoeba vermiformis* strain CCAP 1534/17 18S ribosomal RNA gene, partial sequence (KC188996.1)	PQ345831	100	100
HHFS-6b3	Household	*Vannella miroides* small subunit ribosomal RNA gene, complete sequence (AY183888.1)	PQ345836	95.29	100
HHFS-7a	Household	*Hartmannella vermiformis* 18S ribosomal RNA gene, partial sequence (AY680840.1)	PQ345830	100	100
HHFS-7a2	Household	*Stenamoeba polymorpha* 18S ribosomal RNA gene, partial sequence (KU955320.1)	PQ345829	100	95
HHFS-7b	Household	*Stenamoeba amazonica* strain P119 small subunit ribosomal RNA gene, partial sequence (GU810184.1)	PQ345828	99.22	100
HHFS-8a	Household	*Hartmannella vermiformis* 18S ribosomal RNA gene, partial sequence (AY680840.1)	PQ345827	99.82	100
HHFS-2a	Household	*Ptolemeba bulliensis* isolate Sk13-4e 18S ribosomal RNA gene, partial sequence (KJ542108.1)	PQ345835	100	100
HHFS-4c	Household	*Amoebozoa* sp. Tmp4 partial 18S rRNA gene, isolate Tmp4 (*Micriamoeba* tesseris; FN562429.1)	PX102193	98.98	100
HHFS-6b1	Household	*Protacanthamoeba bohemica* small subunit ribosomal RNA gene, complete sequence (AY960120.1)	PQ345834	92.05	100
LRFS-346a	Laboratory room	*Vermamoeba vermiformis* strain CCAP 1534/17 18S ribosomal RNA gene, partial sequence (KC188996.1)	PQ345824	100	100
LRFS-346b	Laboratory room	*Vannella* sp. BOTM 18S ribosomal RNA gene, partial sequence (JQ271728.1)	PQ345838	98.67	100
LRFS-346c	Laboratory room	*Vannella* sp. BOTM 18S ribosomal RNA gene, partial sequence (JQ271728.1)	PQ345837	98.30	100
LRFS-359	Laboratory room	*Vermamoeba vermiformis* strain CCAP 1534/17 18S ribosomal RNA gene, partial sequence (KC188996.1)	PQ345823	100	100
