# Supplementary material for: Isolation and Molecular Identification of Potentially Pathogenic Free‐Living Amoeba in Tap Water Faucets in Quezon City, Philippines
Source: J Parasitol Res. 2026 Jun 9;2026:8388296. doi: 10.1155/japr/8388296 (PMC13248780; doi:10.1155/japr/8388296)
Supplement: Supplementary file 2 — Supporting Information 2 Table S2: Reference FLA sequences used in phylogenetic analyses, retrieved from the NCBI Core Nucleotide Database. [file JAPR-2026-8388296-s001.pdf]

**TABLE S2.** Reference FLA sequences used in phylogenetic analyses, retrieved from the NCBI Core Nucleotide Database.

| Name of isolate | Accession | Classification* | |
|---|---|---|---|
| | | Phylum | Class |
| *Stenamoeba limacina* strain 4692L | GU810183.1 | Discosea | Flabellinia |
| *Stenamoeba polymorpha* | *KU955320.1* | Discosea | Flabellinia |
| *Stenamoeba amazonica* strain P119 | GU810184.1 | Discosea | Flabellinia |
| *Sappinia pedata* strain CDC V564 | EU980613.1 | Discosea | Flabellinia |
| *Sappinia diploidea* | DQ122380.1 | Discosea | Flabellinia |
| *Sappinia* sp. YQ-2015 | KR069108.1 | Discosea | Flabellinia |
| *Vannella epipetala* clone 4 | DQ913097.1 | Discosea | Flabellinia |
| *Vannella miroides* | AY183888.1 | Discosea | Flabellinia |
| *Protacanthamoeba bohemica* | AY960120.1 | Discosea | Centramoebia |
| *Balamuthia mandrillaris* isolate CDC:V630 | JX524850.1 | Discosea | Centramoebia |
| *Balamuthia mandrillaris* isolate V188 | AF477020.1 | Discosea | Centramoebia |
| *Acanthamoeba polyphaga* 18S | DQ013363.1 | Discosea | Centramoebia |
| *Acanthamoeba castellanii* Castellani ATCC 50374 | U07413.1 | Discosea | Centramoebia |
| *Hartmannella vermiformis* | AF426157.1 | Discosea | Echinamoebida |
| *Vermamoeba vermiformis* strain CCAP 1534/16 | KC161965.1 | Tubulinea | Echinamoebida |
| *Hartmannella vermiformis* isolate KWR-1 | AY502959.1 | Tubulinea | Echinamoebida |
| *Hartmannella abertawensis* strain Page 1980 | DQ190241.1 | Tubulinea | Elardia |
| *Saccamoeba limax* | AF293902.1 | Tubulinea | Elardia |
| *Ptolemeba bulliensis* Sk13-4e | KJ542108.1 | Tubulinea | Elardia |

*Based on Adl et al. (2019)
